# Liquid biopsy: a powerful tool to monitor trastuzumab resistance in HER2-positive metastatic gastric cancer

**DOI:** 10.1186/s40880-018-0344-6

**Published:** 2018-12-18

**Authors:** Lin Shen

**Affiliations:** Department of Gastrointestinal Oncology, Key Laboratory of Carcinogenesis and Translational Research (Ministry of Education/Beijing), Peking University Cancer Hospital and Institute, Fu-Cheng Road 52, Hai-Dian District, Beijing, 100142 China

Human epidermal growth factor receptor 2 (HER2) overexpression/amplification affects about 6.1%–23.0% of gastric cancer patients [1, 2]. All major guidelines recommend HER2 testing to guide the selection for trastuzumab treatment in metastatic gastric cancer (mGC) [3], because doublet chemotherapy with trastuzumab significantly improved overall survival (OS) in the crucial trastuzumab for gastric cancer trial (the ToGA trial) [4]. However, the unsatisfactory gain of median progression-free survival (PFS) stresses important issues of intrinsic and acquired resistance to HER2-targeted therapies [4]. Currently, the underlying molecular mechanism of trastuzumab resistance in mGC is still unclear, and the strategies to real time monitor resistance-specific aberrations are urgently needed. As circulating tumor DNA (ctDNA) carries the same genomic alterations that are present in the primary tumor [5, 6], liquid biopsy-based ctDNA profiling offers a unique opportunity for serially monitoring treatment response in a non-invasive manner, and has opened the door for identification the mechanisms of drug resistance. In a study recently published in *Gut*, titled “Liquid biopsies to track trastuzumab resistance in metastatic HER2-positive gastric cancer”, Wang et al. [7] demonstrated for the first time that longitudinal ctDNA sequencing could promisingly predict tumor shrinkage and progression in HER2 + mGC patients under trastuzumab treatment. Based on the detected molecular alterations from the ctDNA profiling, they further dissected various mechanisms of trastuzumab resistance (Fig. 1).

**Fig. 1:**
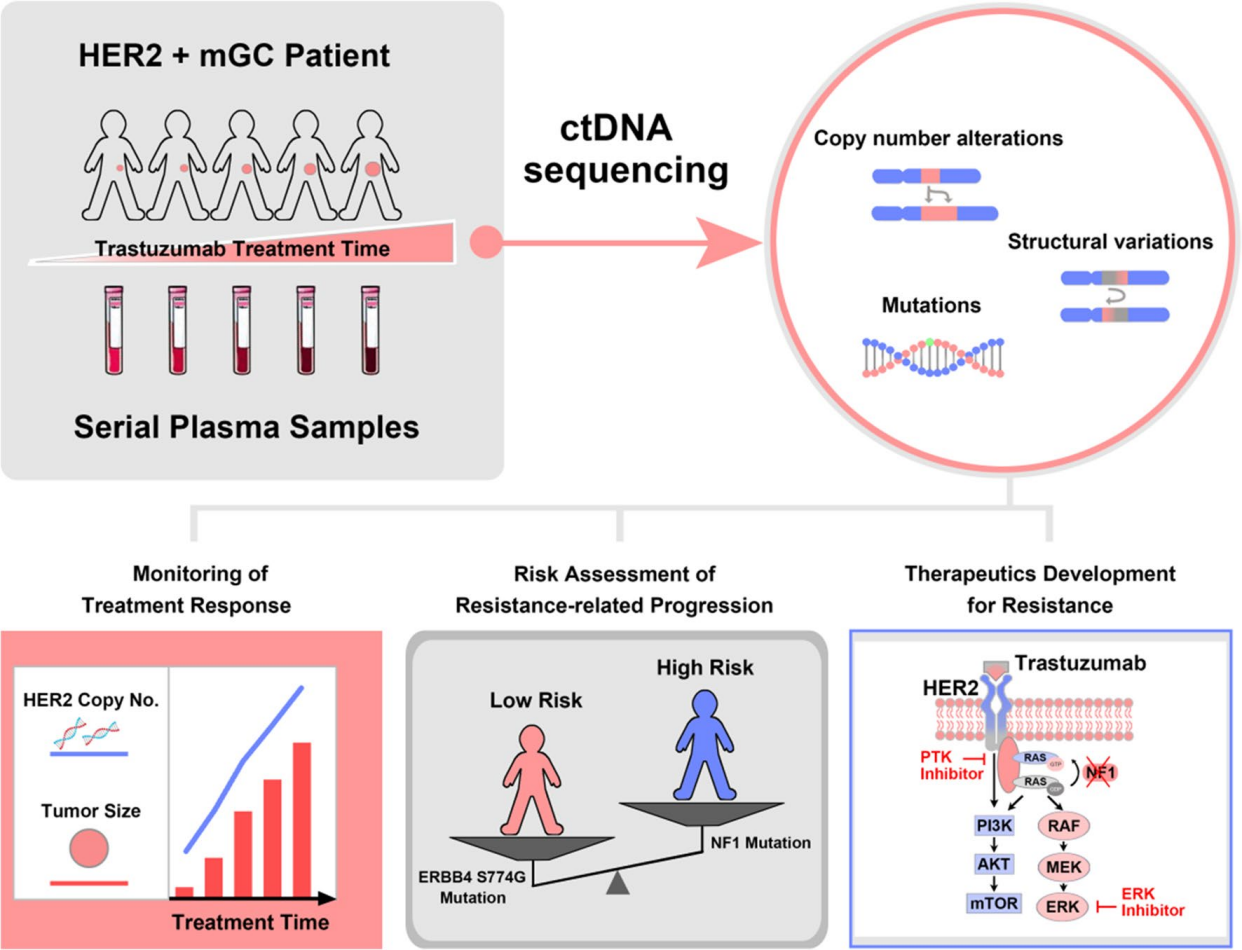
Longitudinal circulating tumor DNA sequencing provides novel insights into gene alterations underlying trastuzumab resistance in HER2-positive metastatic gastric cancer. HER2+: HER2-positive; mGC: metastatic gastric cancer; ctDNA: circulating tumor DNA: Copy No.: copy number

In this study, the authors sequenced 416 clinically relevant genes to determine the genomic profiles of GC patients. The molecular alterations, including copy number alterations, mutations and structural variations, detected in 78 paired plasma and tissue samples (46 from HER2+ patients and 32 from HER2− patients) were used to evaluate the consistency of detection between the “liquid” and “solid” biopsies. As expected, the alterations detected in plasma provided a good representation of the status in the tumor tissues. In particular, HER2 amplification status, the major marker of HER2 + GC, detected by plasma-based ctDNA sequencing was highly consistent with that by tissue-based sequencing and fluorescence in situ hybridisation. These findings indicated that plasma-based ctDNA profiling might provide insights into HER2 amplification in HER2 + GC.

Further longitudinal analysis were perform in 97 time-course plasma samples collected from 24 HER2 + mGC patients to track the resistance during trastuzumab treatment. As compared to plasma carcinoembryonic antigen, the copy number variations (CNVs) of *HER2* in ctDNA were better at tracking trastuzumab treatment response. Generally, the *HER2* copy number decreased during treatment compared with baseline and levels of progressive disease (PD), indicating tumor shrinkage. Additionally, most patients with intrinsic resistance-related progression presented higher *HER2* copy numbers at PD than baseline, while the patients with acquired resistance often had lower HER2 copy numbers at PD.

From the same ctDNA profiles, the authors found evidence for reported mechanisms of trastuzumab resistance in 76.5% patients. They found that the patients with multiple resistance mechanisms (*PIK3CA/R1/C3* and *ERBB2/4* mutations) displayed much shorter PFS during trastuzumab treatment. These findings suggested that liquid biopsy-based ctDNA profiling can effectively capture the heterogeneity information about acquired resistance. Furthermore, they also observe several novel resistance mechanisms. One of them is the *ERBB4* S774G mutation. This novel mutation can increase the sensitivity to trastuzumab treatment, suggesting for the first time that the mutation of *ERBB4* play a critical role in mediating resistance to ERBB2 inhibitors in HER2 + GC. Another novel resistance mechanism is the emergence of *NF1* mutations. Using in vitro and in vivo experiments, the authors demonstrated that *NF1* was a resistance-related gene and could be used as a potential resistance marker of trastuzumab. *NF1* lost (mutation or deletion) contributes to driving resistance of HER2-targeted therapy via elevating pMEK/ERK activation, whereas the combination of HER2 and MEK/ERK inhibitors might overcome trastuzumab resistance.

This study has some obvious limitations. First, other rare genomic or even nongenomic mechanisms of trastuzumab resistance may have been missed by the 416-gene panel. Second, the relatively small number of patient enrolled does not allow drawing definitive conclusion on the predictive value of this liquid biopsy approach. Nevertheless, the study provides helpful information to monitor the treatment response of trastuzumab and develop therapeutic strategies for the HER2 + mGC patients with trastuzumab resistance.

## Abbreviations

HER2: human epidermal growth factor receptor 2
mGC: metastatic gastric cancer
OS: overall survival
ToGA: trastuzumab for gastric cancer
PFS: progression-free survival
ctDNA: circulating tumor DNA
CNV: copy number variation
PD: progressive disease
PIK3CA/R1/C3: phosphoinositide 3-kinase catalytic subunit alpha/regulatory subunit 1/catalytic subunit type 3
ERBB2/4: receptor tyrosine-protein kinase erbB-2/4
MEK/ERK: mitogen-activated protein kinase kinase/extracellular signal-regulated kinases
